# Novel Alpha-, Beta-, and Gammaherpesviruses in Neotropical Carnivores of Brazil

**DOI:** 10.1155/2024/1347516

**Published:** 2024-06-06

**Authors:** Ana Carolina Ewbank, José Luiz Catão-Dias, Pedro Enrique Navas-Suarez, Aricia Duarte-Benvenuto, Roberta Zamana-Ramblas, Eduardo Ferreira-Machado, Henrique Christino Lial, Pablo Ibáñez-Porras, Irene Sacristán, Carlos Sacristán

**Affiliations:** ^1^ Faculdade de Medicina Veterinária e Zootecnia Universidade de São Paulo Av. Prof. Orlando Marques de Paiva, 87-Butantã, São Paulo05508-270SPBrazil; ^2^ Centro de Investigación en Sanidad Animal (CISA-INIA) Spanish National Research Council (CSIC) Carretera Algete-El Casar de Talamanca, Km. 8,1, 28130, Valdeolmos28130Spain

## Abstract

The knowledge regarding infectious agents affecting wildlife is crucial for species' conservation. We hypothesized that herpesviruses are present in wild Neotropical carnivores. Herein, we used DNA polymerase and glycoprotein B broad-spectrum PCRs to molecularly survey the presence of herpesviruses in spleen and/or lung samples of 53 wild Neotropical carnivores of Brazil, comprising the families Canidae, Felidae, Mustelidae, and Procyonidae. The percentage of PCR-positives was 28.3% (15/53). An alphaherpesvirus was found in a Neotropical river otter (*Lontra longicaudis*, 1/1), a betaherpesvirus in a lesser grison (*Galictis cuja*, 1/3), and different gammaherpesviruses in Neotropical river otter (1/1), lesser grison (1/3), crab-eating raccoons (*Procyon cancrivorus*, 8/9), South American coati (*Nasua nasua*, 1/2), southern tiger cat (*Leopardus guttulus*, 1/2), jaguarundi (*Puma yagouaroundi*, 1/5), and ocelot (*Leopardus pardalis*, 1/10). None of the tested canids were herpesvirus-positive. This is the first report of herpesvirus in procyonids, and in jaguarundi, southern tiger cat, lesser grison, and Neotropical river otter. This study broadens the host range of herpesviruses in Neotropical carnivores.

## 1. Introduction

Herpesviruses (order *Herpesvirales*) are double-stranded DNA viruses of over 120-kilobase, able to establish latency in their natural host [1]. The *Orthoherpesviridae* family is divided into subfamilies *Alphaherpesvirinae*, *Betaherpesvirinae*, and *Gammaherpesvirinae* [1]. Most of the herpesvirus infections are mild in immunocompetent natural hosts; however, severe disease has been observed in immunosuppressed or nonadapted hosts, or in individuals coinfected with other agents [2, 3].

The order Carnivora comprises 15 different families (https://www.itis.gov); among them, herpesviruses have been described in Felidae, Canidae, Mustelidae, Hyaenidae, Ursidae, Odobenidae, Otariidae, and Phocidae [4, 5, 6, 7, 8, 9, 10, 11, 12].

In Felidae, the most studied herpesvirus species are *Felid alphaherpesvirus 1* (genus *Varicellovirus*) and *Felid gammaherpesvirus 1* (genus *Percavirus*), which have the domestic cat (*Felis catus*) as their natural host [6, 7, 13, 14]. In cats, *Felid alphaherpesvirus 1* causes upper respiratory tract (rhinotracheitis) and ocular disease, and even pneumonia and nonsuppurative meningoencephalitis, and the infection is often fatal in kittens [15, 16, 17]. The pathogenicity of *Felid gammaherpesvirus 1* is unknown [18]. In wild felids, *Felid alphaherpesvirus 1* has been molecularly detected in free-ranging and captive snow leopards (*Panthera uncia*) of Mongolia and China, respectively, causing disease and likely death in the former animals [19, 20], and in a captive tiger (*Panthera tigris*) of China with upper respiratory disease [21], and several other tigers from Chinese zoos [22]. This pathogen is endemic in captive cheetah (*Acinonyx jubatus*) from North America and linked to mortality [23]. Of note, vaccination against *Felid alphaherpesvirus 1* can also cause disease [23]. *Felid gammaherpesvirus 1* has been recently found in a wild felid species, i.e., the leopard cat (*Prionailurus bengalensis*) of Japan, without apparent associated lesions or clinical signs [24]. Additionally, other herpesviral species that likely have wild felids as natural hosts have been described: (i) Panthera leo gammaherpesvirus 1 in a free-ranging African lion (*Panthera leo*) of Tanzania [5]; (ii) Puma concolor gammaherpesvirus 1 in pumas (*Puma concol*or) of the US [6]; (iii) Lynx rufus gammaherpesvirus 1 in bobcats (*Lynx rufus*), pumas and a Canada lynx (*Lynx canadensis*) of the US [6, 14, 25, 26]; (iv) Lynx rufus gammaherpesvirus 2 in bobcats of the US [6, 25]; (v) Leopardus pardalis gammaherpesvirus 1 in ocelots (*Leopardus pardalis*) of Panama [25]; and (vi) Lynx canadensis gammaherpesvirus 1 in Canada lynxes (*Lynx canadensis*) of the US and Canada [14]. No associated lesions were described in any of these reports.

Regarding canids, *Canine alphaherpesvirus 1* (genus *Varicellovirus*) has dogs (*Canis lupus familiaris*) as the natural host and can cause severe necrotizing hemorrhagic disease and even death in puppies, especially in 1–2 weeks old puppies, and asymptomatic infection in older puppies and adults, although occasionally the virus causes mild respiratory disease, reproductive disorders, and genital lesions [4]. This virus also causes clinical disease in experimentally inoculated red foxes (*Vulpes vulpes*) [27] and was recently detected in free-ranging coyotes (*Canis latrans*) with no description regarding pathogenicity [28]. In wild canids, a specific herpesvirus was described in Darwin's foxes (*Lycalopex fulvipes*) of Chile, the Darwin's fox gammaherpesvirus, with no associated lesions [29].

In mustelids, alphaherpesviruses have been found in Eurasian badger (*Meles meles*) and American marten (*Martes americana*), and betaherpesvirus in American marten, all without associated lesions [11, 28]. In contrast, although no clinical disease was described for gammaherpesviruses reported in Eurasian badger, oriental small-clawed otter (*Aonyx cinerea*), and American marten, these viruses have been detected in oral lesions and nasal secretions in sea otters (*Enhydra lutris*), cutaneous lesions in fisher (*Martes pennanti*), and neoplasms and neurological disease in European mink (*Mustela lutreola*) [11, 30, 31, 32]. Nevertheless, it is unclear if the reported lesions were caused by herpesviruses.

In Ursidae, a gammaherpesvirus (Ursid herpesvirus 1) was detected in captive sun bears (*Helarctos malayanus*) with and without oral squamous cell carcinomas [33, 34] and two additional gammaherpesviruses in several black bears (*Ursus americanus*) [9]. Finally, in Hyaenidae, Crocuta crocuta gammaherpesvirus 1 was found in a free-ranging spotted hyena (*Crocuta crocuta*) of Tanzania [5].

On the other hand, there are several reports of herpesvirus of noncarnivorous domestic animals affecting wildlife. The *Suid alphaherpesvirus 1* (*Varicellovirus*), which naturally infects suids and causes pseudorabies, has been reported in Iberian lynxes (*Lynx pardinus*), pumas, coyotes, wolves, red foxes, Artic foxes (*Vulpes lagopus*), brown bears (*Ursus arctos*), a black bear, minks, and northern raccoons (*Procyon lotor*), with associated pathogenicity in all these species (e.g., encephalitis and pruritus) [35, 36, 37, 38, 39, 40, 41, 42, 43, 44, 45]. *Equine alphaherpesvirus 1* has been detected in captive black bears and captive polar bears (*Ursus maritimus*) with encephalitis [46, 47], and *Equine herpesvirus 9* reportedly caused encephalitis in a captive polar bear [48, 49]. Of note, *Bovine alphaherpesvirus 4* may also infect cats, although its pathogenicity is still unclear [50]. Finally, an alphaherpesvirus of the genus *Simplexvirus*, with human origin, reportedly caused encephalitis in a skunk (*Mephitis mephitis*) [51].

In spite of the above, the current knowledge on herpesviruses in Neotropical carnivores is scarce [29], limited to the infection of Darwin's foxes by a novel gammaherpesvirus [29] in Chile, and of *Felid alphaherpesvirus 1* in two jaguars (*Panthera onca*, one captive and one wild) and a wild oncilla (*Leopardus tigrinus*) in Brazil, with no information regarding pathogenicity [52]. We hypothesized that novel herpesviruses are present in wild Neotropical carnivores. Our goal was to molecularly survey the infection of herpesviruses in wild Neotropical carnivores of Brazil.

## 2. Materials and Methods

### 2.1. Samples

**Figure 1:**
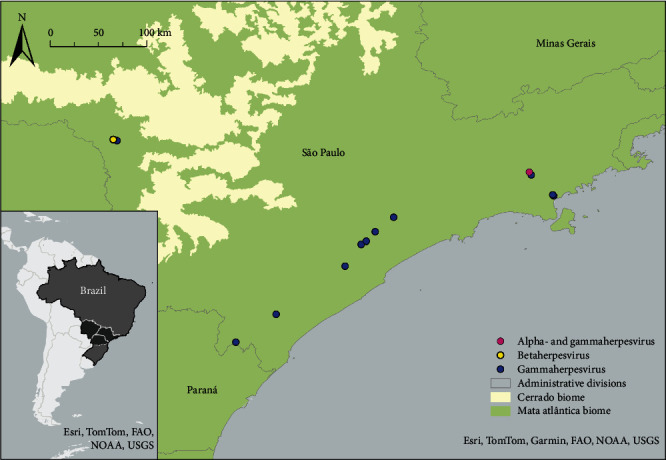
Brazilian states of origin where the tested carnivores were samples—marked in dark grey in the map of Brazil—and positive cases to beta (yellow dot), gammaherpesviruses (blue dot), or coinfected with alpha- and gammaherpesviruses (pink dot).

We analyzed spleen and/or lung samples of 53 carnivores of the following species: crab-eating fox (*Cerdocyon thous*, *n* = 10), maned wolf (*Chrysocyon brachyurus*, *n* = 6), bush dog (*Speothos venaticus*, *n* = 1), ocelot (*n* = 10), jaguarundi (*Puma yagouaroundi*, *n* = 5), puma (*n* = 4), southern tiger cat (*Leopardus guttulus*, *n* = 2), lesser grison (*Galictis cuja*, *n* = 3), Neotropical river otter (*Lontra longicaudis*, *n* = 1), crab-eating raccoon (*Procyon cancrivorus*, *n* = 9), and South American coati (*Nasua nasua*, *n* = 2). These species are comprised in the families Canidae (crab-eating fox, maned wolf, and bush dog), Felidae (jaguarundi, ocelot, northern tiger cat, and puma), Mustelidae (lesser grison and Neotropical river otter), and Procyonidae (South American coati and crab-eating raccoon). The animals were road-killed (*n* = 50), euthanized (*n* = 1), dead after 2 days under rehabilitation (*n* = 1), or were found dead (*n* = 1). The samples were preserved at −20°C until tested. The animals were found in the states of Santa Catarina (*n* = 1), Paraná (*n* = 2), São Paulo (*n* = 46), Minas Gerais (*n* = 1), and Mato Grosso do Sul (*n* = 2), Brazil (Figure 1). Detailed information is displayed in Table 1. Some of these cases were previously tested for adenovirus by nested PCR [53]. All procedures were in full compliance and approved by the Biodiversity Information and Authorization System (SISBIO 58745), Brazilian Ministry of Environment, and by the Ethical Committee of the School of Veterinary Medicine and Animal Sciences, University of São Paulo (Ceuavet n° 7198020317).

### 2.2. Molecular Analyses

After manual (with individualized scalpel blades in sterilized Petri dishes, using one blade and dish per sample) or mechanical homogenization (with two steel beads of 2.4 mm (Omni international, Kennesaw, GA, USA), 100 *µ*l of PBS 1x, 180 *µ*l of buffer ATL (Qiagen, Hilden, Germany), and in an Omni Bead Ruptor 12 homogenizer using two cycles of 60” at 6 m/s, with an interval of 30” between them (Omni international) of 25 mg frozen spleen and lung samples, total DNA was extracted with the DNeasy Blood & Tissue kit (Qiagen), according to the manufacturer's instructions. Subsequently, the DNA samples were tested with broad-spectrum nested endpoint PCR protocols to amplify a 230–330 bp fragment of the DNA polymerase (DPOL) gene using an annealing temperature of 46°C and a 500 bp fragment of glycoprotein B (gB) gene using an annealing temperature of 46°C, according to VanDevanter et al. [54] and Ehlers et al. [5], as described by Novoselecki et al. [55]. All the amplicons of the expected size were purified with ExoSAP-IT (Affymetrix, Santa Clara, CA, USA) and directly sequenced in both directions in an ABI 3730 DNA analyzer (Life Technologies—Applied Biosystems, Foster City, CA), using the BigDye Terminator v3.1 Cycle Sequencing kit. Consensus sequences were constructed using Mega 7.0. In those cases that had lung and/or liver samples DPOL—and/or gB-PCR-positive, all remaining tissue samples were also extracted and tested with the PCR protocols described above (Table S1).

After a nucleotide Blast search (https://blast.ncbi.nlm.nih.gov/Blast.cgi, as of March 2023), we used Mega 7.0 [56] to calculate the *P* distance among the obtained sequences and the closest ones from GenBank/EMBL/DDBJ. DPOL and gB Maximum-likelihood phylograms were constructed with Mega 7.0 after the ClustalW alignment of the DPOL (55–61 amino acids) and gB herpesvirus (151 amino acids) sequences obtained in this study, the closest ones from GenBank, and representative herpesviral species accepted by the International Committee on Taxonomy of Viruses (ICTV) until March 2023. A *Human alphaherpesvirus 3* sequence was used as outgroup for gB phylogram. Prior to the construction of the amino acid maximum-likelihood DPOL and gB phylogenetic trees, we used the program ProtTest 3.4.2 [57] to select the best evolution model, based on the Akaike information criterion.

### 2.3. Statistical Analysis

Differences in herpesvirus prevalence between the independent variables (sex, age, body condition, season (dry or rain), activity (diurnal or nocturnal), ambient (field, forest, or river), and diet (carnivore or omnivore)) have been evaluated trough chi-square and Kruskal–Wallis test Dunnett's post-test with a significance of *p*  < 0.05, using GraphPad Prism 5.0 (GraphPAD Software Inc., La Jolla, CA, USA).

### 2.4. Histopathological Analysis

All herpesvirus-positive carnivores were analyzed by histopathology. Representative tissue samples were fixed in 10% buffered formalin, embedded in paraffin, cut at 5 *µ*m, and stained with hematoxylin and eosin.

## 3. Results

### 3.1. Molecular Analysis

The percentage of positive animals was 28.3% (15/53), including 81.8% (9/11) of the tested procyonids, 75% (3/4) of the mustelids, and 14.3% (3/21) of the felids, while none of the 17 tested canids were positive. The species that tested herpesvirus-positive were as follows: crab-eating raccoon (8/9) and South American coati (1/2) from Procyonidae family; lesser grison (2/3) and Neotropical river otter (1/1) from Mustelidae family and southern tiger cat (1/2), and jaguarundi (1/5) and ocelot (1/10) from Felidae family (Figure 1, Table S1). Most of the herpesvirus-positive cases (14 out of 15) had good nutritional condition, and one had regular nutritional condition. Additional details about the herpesvirus positive cases are displayed in Table S1.

Regarding the detection PCR technique, adequate gB sequences were amplified in 14 individuals (eight crab-eating raccoons, one South American coati, one lesser grison, one Neotropical river otter, one southern tiger cat, one jaguarundi, and one ocelot); while adequate DPOL sequences were retrieved from 10 individuals (six crab-eating raccoons, one South American coati, one lesser grison, one Neotropical river otter, and one ocelot). Detailed results, including the herpesvirus-positive tissues with DPOL and gB PCR protocols, and the percentage of identity to the closest herpesviruses available at GenBank/DDBJ/EMBL database are shown in Table 2 and Table S1.

Overall, 8 out of 47 spleen samples were herpesvirus-positive, and 12 out of 40 lung samples. All the herpesviruses sequenced from additional tissue samples (Table S1) were identical to the ones found in the lung and/or spleen of the host species, except for the South American coati RK314, which presented two DPOL nucleotide and amino acid sequence types (in kidney and spinal cord, respectively). Representative gB consensus sequences of different gammaherpesviruses were submitted to GenBank under accession numbers OQ926578 (procyonid gammaherpesvirus 1, identified in crab-eating raccoons), OQ926579 (procyonid gammaherpesvirus identified in a South American coati RK314), OQ926580 (identified in the ocelot RK175), OQ926581 (found in the southern tiger cat RK303), OQ926582 (found in the jaguarundi RK300), OQ926583 (detected in the Neotropical river otter RK35), and OQ926584 (detected in the lesser grison RK12). Representative DPOL sequences were submitted to GenBank under accession numbers OQ980212 (procyonid gammaherpesvirus 1, from a crab-eating raccoon), OQ980213 (procyonid gammaherpesvirus 2, from the spinal cord of the South American coati RK314), OQ980214 (procyonid gammaherpesvirus 3, from the kidney of the South American coati RK314), OQ980215 (Leopardus pardalis gammaherpesvirus from the ocelot RK175), OQ980216 (gammaherpesvirus from a Neotropical river otter RK35), OQ980217 (alphaherpesvirus from the Neotropical river otter RK35), and OQ980218 (gammaherpesvirus from the lesser grison RK284).

**Figure 2:**
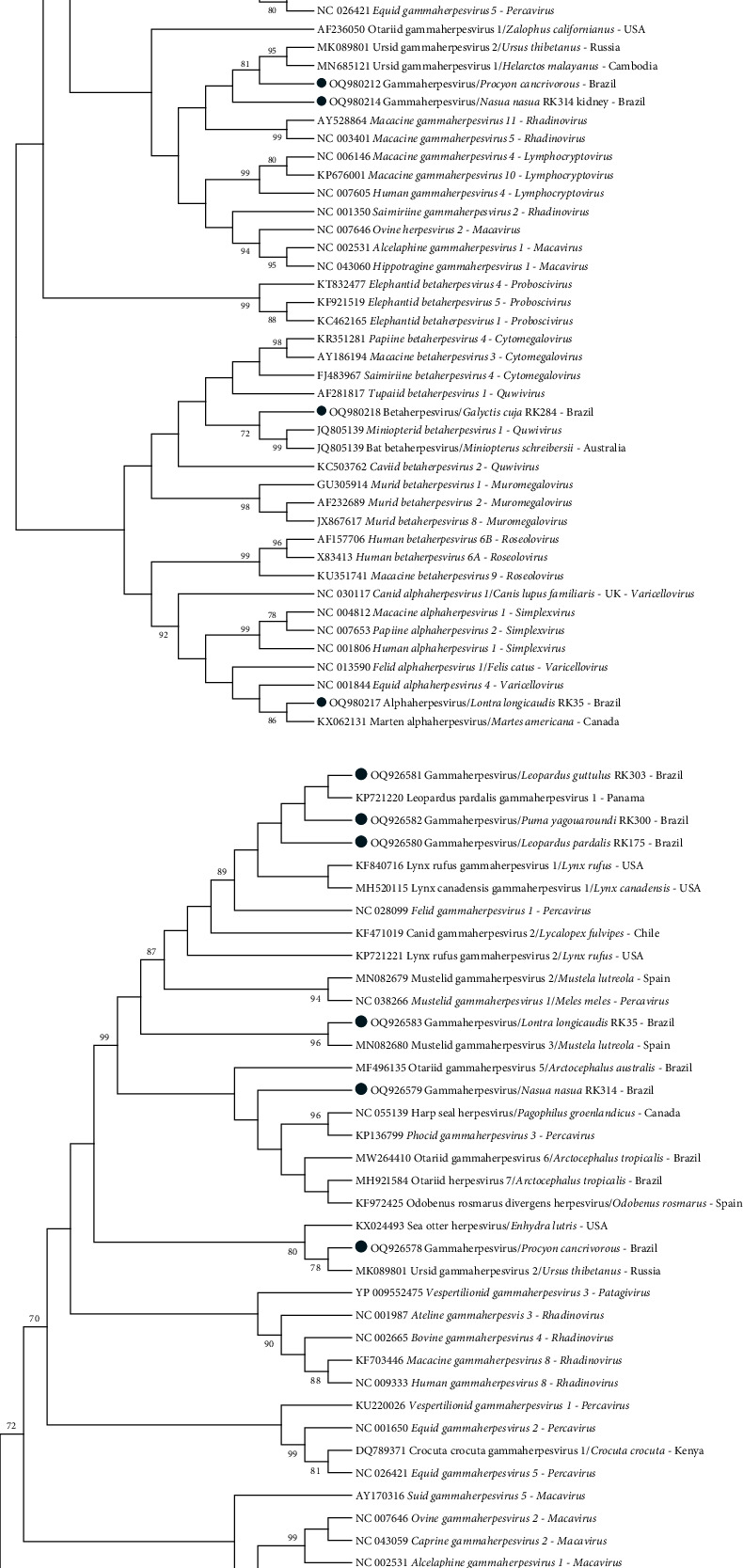
Maximum-likelihood phylograms of (a) the alignment of the deduced DNA polymerase (DPOL) amino acid (aa) sequences (55–61 amino acids) obtained in this study (blue dots), representative DPOL sequences of herpesviral species accepted by the International Committee on Viral Taxonomy, and herpesviral DPOL sequences previously described in carnivores and (b) the alignment of the deduced glycoprotein B (gB) aa sequences (151 aa) obtained in this study (blue dots), representative gB sequences of herpesviral species accepted by the International Committee on Viral Taxonomy, and herpesvirus gB sequences previously described in carnivores. *Human alphaherpesvirus 3* was selected as outgroup for glycoprotein B phylogram. 1,000 bootstrap replications were selected for both phylograms. Bootstrap values lower than 70 were omitted. The DNA polymerase and glycoprotein B phylograms were based on LG + I + G and LG + G models, respectively.

Regarding the herpesvirus subfamilies, herein we detected (i) an alphaherpesvirus in a Neotropical river otter, (ii) a betaherpesvirus in a lesser grison, and (iii) eight different gammaherpesvirus sequence types in an ocelot, a jaguarundi, a southern tiger cat, a lesser grison, a Neotropical river otter (coinfected with an alphaherpesvirus), eight crab-eating raccoons, and a South American coati (the coati presented two different sequence types, in kidney and in spinal cord, with nt and amino acid identities of 66.7% and 57.8% between them) (Table S1). The nt gB sequences obtained in the felids were highly similar among them (99.1% ocelot vs. jagoarundi, 98.9% ocelot vs. southern tiger cat, 98.9% jaguarundi vs southern tiger cat), and 100% identical among them when translated to amino acid. According to the DPOL phylogram, the gammaherpesvirus was found in an ocelot cluster with other sequences within the genus *Percavirus*, as well as those obtained in a neotropical river otter and in the spinal cord of a South American coati (Figure 2(a)). The gammaherpesviruses found in crab-eating raccoons and in the kidney of a South American coati were not assigned to any genus. The betaherpesvirus was obtained in a lesser grison cluster with betaherpesviruses from bats, including a sequence of the genus *Quwivirus*. Finally, the alphaherpesvirus sequence retrieved from a Neotropical river otter grouped with a sequence from another mustelid—an American marten—but was not correctly assigned to any genus (Figure 2(a)). According to the gB phylogenetic tree (Figure 2(b)), the gammaherpesvirus sequences found in felids, Neotropical river otter, and South American coati were classified into the genus *Percavirus*, while the gammaherpesvirus from crab-eating raccoon clusters with Ursid gammaherpesvirus 2 (MK089801) and sea otter herpesvirus (KX024493), without an assigned genus; all the gammaherpesviruses from felids were grouped in the same subclade (Figure 2(b)).

### 3.2. Statistical Analysis

There were no statistically significant differences for any of the independent variables analyzed (Table S2).

### 3.3. Histopathological Analysis

None of the herpesvirus-positive animals presented histological lesions consistent with those attributed to these agents.

## 4. Discussion

To the authors' knowledge, this is the largest herpesvirus survey based on broad-spectrum PCRs encompassing different carnivore species. In procyonids, eight crab-eating raccoons and one South American coati were gammaherpesvirus-positive. To our best knowledge, there are no previous herpesvirus descriptions in the family Procyonidae. The deduced amino acid gammaherpesviruses described in crab-eating raccoons and a South American coatis presented identities below 95.5% for highly preserved genes (DPOL and gB) when compared to the closest sequences from GenBank, and were found in novel host species, which supports its classification as novel gammaherpesvirus species, as described by Ewbank et al. [58]. Based on this, the crab-eating raccoon likely presented one novel gammaherpesvirus species, and the South American coati two novel gammaherpesvirus species (in spinal cord and kidney, based on DPOL analysis). The proposed common names for them are procyonid gammaherpesvirus 1 for the one found in crab-eating raccoons, and procyonid gammaherpesvirus 2 and 3 for those amplified in the spinal cord and kidney of a South American coati. Moreover, the detection of procyonid gammaherpesvirus 1 in eight raccoons from different regions of the state of São Paulo suggests that this species could be the natural host of that gammaherpesvirus [55, 59].

In felids, the DPOL gammaherpesvirus nucleotide sequence described in the ocelot from Brazil was identical to the Leopardus pardalis gammaherpesvirus 1 sequence (KP721220) found in two animals of the same species in Panama [25], which belong to populations separated by more than 4,800 km; this suggests the role of ocelots as a natural host for this virus. Additionally, three gammaherpesvirus gB sequence types were found in the ocelot mentioned above, and in a southern tiger cat and a jaguarundi, the two latter species without previous descriptions of herpesvirus; these gB nucleotide sequences presented identities of 99.6%, 98.9%, and 99.1% to a Leopardus pardalis gammaherpesvirus 1 sequence (KP721220), respectively. The deduced amino acid gB sequences from our felids were 100% identical among them, to the Leopardus pardalis gammaherpesvirus (KP721220) and to the *Felid gammaherpesvirus 1* sequences (LC198234, KF840715, KP721220, NC_028099) found in cats from Japan and the USA. We aligned our gB and DPOL with a 3,476 bp fragment of Leopardus pardalis gammaherpesvirus 1 comprising both genes described by Lozano et al. [25], and they perfectly overlap. In order to elucidate if there are differences in the conservation of DPOL and gB sequences, we compared the DPOL and gB complete sequences available for an ocelot of Panama (KP721220) and the *Felid gammaherpesvirus 1* (KF840715, NC_028099) and observed that DPOL gene was less conserved (similarities of 90.8% nt and 93.4% aa) than the gB gene (similarities of 93.4% and 99.1%). The same was observed in gammaherpesviruses from river dolphins [55]. Based on this and our identity analyses, we likely detected the Leopardus pardalis gammaherpesvirus 1 in our ocelot, nor the *Felid gammaherpesvirus 1*. This classification in two separate species was also done by Lozano et al. [25] when compared fragments of 3,476 bp Leopardus pardalis gammaherpesvirus 1 and *Felid gammaherpesvirus 1*. The gammaherpesviruses detected in southern tiger cat and a jaguarundi could also be novel species, but the higher preservation of gB gene and the lack of DPOL sequences do not allow an adequate characterization. A limitation of our study is that we were not able to obtain longer fragments, which would further improve the phylogenetic resolution. Nevertheless, our findings broaden the host range of herpesviruses in felids present in the Americas. Gammaherpesviruses of wild felids have been described in each wild felid species in northern North America (i.e., puma, Canada lynx, and bobcat) [14], and in an Ocelot from Central America [25], but to the authors' knowledge, this is the first gammaherpesvirus report in South American wild felids, in spite of the high diversity of felid species in the region. In South American wild felids, the only herpesviruses known to this date was *Felid alphaherpesvirus 1*, described before in two jaguars (one captive and one free-ranging) and a captive oncilla of Brazil [52]. This agent is also present in domestic cats of that country, its natural host species [60]. Of note, none of the studied felids were positive to *Felid alphaherpesvirus 1*.

The findings of an alpha- and a gammaherpesvirus in a Neotropical river otter (RK35), and of a betaherpesvirus in lesser grison (RK284) and a gammaherpesvirus in another grison (RK12), widen the host range of herpesviruses in mustelids. To our best knowledge, only a betaherpesvirus has been previously described in mustelids—also in carnivores—specifically in American marten from Canada [28], while several alpha- and gammaherpesviruses have been described in mustelids [11, 28].

None of the tested canids were herpesvirus-positive, in spite of they were the most numerous groups in our study (*n* = 17), with 51 tissue samples analyzed with two different protocols. Of note, aside from *Canine alphaherpesvirus 1*, only another herpesvirus has been described in wild canids, the Darwin's fox gammaherpesvirus in Darwin's foxes of Chile [29]. Larger studies, including most canid specimens of each species and tissue types, are required to establish if this group hosts novel herpesvirus species in Brazil, as observed in Darwin foxes from Chile [29].

None of the herpesvirus-positive animals presented histopathological lesions consistent with herpesviral disease. In carnivores, some alphaherpesviruses have been associated with upper respiratory tract (*Felid alphaherpesvirus 1*) and necrotizing hemorrhagic disease (*Canine alphaherpesvirus 1*) [4, 17]. Regarding gammaherpesviruses, genital reactivation of *Mustelid gammaherpesvirus 1* has been associated with absorption of fetuses, abortion, and poorer body condition in Eurasian badgers [59, 61], and some other gammaherpesvirus species have been associated with lymphoproliferative disorders, neoplasms, and skin lesions in pinnipeds [8]. Finally, the pathogenicity of betaherpesviruses, if any, is largely unknown in this order. The pathogenic potential of these herpesviruses should be studied in the future, especially in calves, which are usually not included in the studies and may suffer more virulent infections, higher morbidity, and mortality, as reported for *Felid alphaherpesvirus 1* and *Canine alphaherpesvirus 1* in domestic carnivores [4, 17]. Moreover, it is important to remark that herpesviruses can reactivate under immunosuppressive conditions (e.g., concomitant disease, intoxication by chemical pollutants) [2, 62]. In our study, most of the animals presented good nutritional condition. Gammaherpesviruses were detected in spleen samples of an ocelot and six crab-eating raccoons, likely because of the presence of the viruses in lymphocytes—the cells in which they establish latency [63]—and in nine lung samples, also a highly vascularized tissue, and was not possible to differentiate latency from primoinfection or reactivation in these tissues. A betaherpesvirus was found in the lung of a lesser grison; the latency of viruses within the subfamily *Betaherpesvirinae* has been reported in CD34+ hematopoietic stem cells, CD14+ monocytes, T lymphocytes, and maybe in neuronal cells [63], and we are not able to discard that our virus was latent in any of these circulating blood cells. The presence of an alphaherpesvirus in the spleen sample of a Neotropical river otter may indicate viremia, as these viruses establish their latency in sensory neuron cells [63].

## 5. Conclusions

The knowledge about infectious agents is essential for species conservation [64]. This study contributes to our understanding of carnivore herpesviruses evolutionary history. This study broadens the host range of herpesviruses in Neotropical carnivores, including the description of novel host—families Felidae (jaguarundi and southern tiger cat), Mustelidae (lesser grison and Neotropical river otter), and Procyonidae (crab-eating raccoons and South American coati), and viral species. The pathogenicity of these viruses should be further investigated.

## Figures and Tables

**Table 1:** Family, species, and number of carnivores tested for herpesvirus.

Family	Species	*n*	Sex			Age class		Body condition		
			M	F	C	J	A	R	G	U
Canidae	Crab-eating fox (*C. thous*)	10	5	5	1	1	8	1	9	—
	Maned wolf (*C. brachyurus*)	6	3	3	—	1	5	3	3	—
	Bush dog (*S*. *venaticus*)	1	1	—	—	—	1	—	—	1

Felidae	Ocelot (*L*. *pardalis*)	10	8	2	—	3	7	1	9	—
	Jaguarundi (*P. yagouaroundi*)	5	5	—	—	2	3	1	4	—
	Southern tiger cat (*L. guttulus*)	2	1	1	—	2	—	—	2	—
	Puma (*P*. *concolor*)	4	2	2	—	3	1	1	3	—

Mustelidae	Lesser grison (*G*. *cuja*)	3	3	—	—	1	2	1	2	—
	Neotropical river otter (*L*. *longicaudis*)	1	1	—	—	—	1	—	1	—

Procyonidae	Crab-eating raccoon (*P*. *cancrivorus*)	9	6	3	—	4	5	—	9	—
	South American coati (*N. nasua*)	2	1	1	—	—	2	—	2	—
	*Overall*	53	36	17	1	17	35	8	44	1

*R* = regular, *G* = good, *U* = undefined.

**Table 2:** Nucleotide (nt) and amino acid (aa) identities of the DNA polymerase and glycoprotein B herpesviral consensus sequences obtained from the positive carnivores when compared to the closest ones from GenBank/EMBL/DDBJ database.

Species	DPOL sequence amplified in our study	DPOL identities		gB sequence amplified in our study	gB identities	
		Closest nt sequence	Closest aa sequence		Closest nt sequence	Closest aa sequence
Southern tiger cat (*L. guttulus*)	ND	—	—	OQ926581	98.9% to our ocelot gammaherpesvirus and to a Leopardus pardalis gammaherpesvirus (KP721220) found in a ocelot of Panama; 93.6% to Felis catus gammaherpesvirus sequences (LC198234, KF840715, NC_028099) found in cats from Japan and USA	The aa gB sequences found in southern tiger cat, jaguarondi and ocelot were identical among them, and to a Leopardus pardalis gammaherpesvirus from Panama (KP721220) and Felis catus gammaherpesvirus sequences (LC198234, KF840715, NC_028099) found in cats from Japan and USA

Jaguarundi (*P. yagouarandi*)	ND	—	—	OQ926582	99.1% to our ocelot gammaherpesvirus and to a Leopardus pardalis gammaherpesvirus (KP721220) found in a ocelot of Panama; 93.6% to Felis catus gammaherpesvirus sequences (LC198234, KF840715, NC_028099) found in cats from Japan and USA	

Ocelot (*L. pardalis*)	OQ980215	100% to Leopardus pardalis gammaherpesvirus 1 (KP721220); 88.5% to Felid gammaherpesvirus 1 sequences (KT595939, NC_028099) of the US	100% to Leopardus pardalis gammaherpesvirus 1 (KP721220, our sequence has four more amino acids), 90.6% Felid gammaherpesvirus 1 (KT595939, NC_028099) of the same length of the US	OQ926580	99.6% to a Leopardus pardalis gammaherpesvirus (KP721220) found in a ocelot of Panama; 93.6% to Felid gammaherpesvirus 1 sequences (LC198234, KF840715, NC_028099) found in cats from Japan and USA	

Lesser grison (*G. cuja*)	OQ980218	Case RK284: 65.6% to a betaherpesvirus of a Sumatran greater bamboo bat (*Tylonycteris robustula*, JQ814846)	Case RK284: 60.9% to a betaherpesvirus of a Schreibers's long-fingered bat (*Miniopterus schreibersii*, JQ805139)	OQ926584	Case RK12: 89.5% similar to Harp seal gammaherpesvirus (NC_055139)	Case RK12: 96.3% similar to Otariid gammaherpesvirus 5 (MF496135, MH921583)

Neotropical river otter (*L. longicaudis*)	OQ980216 (in lung) OQ980217 (in spinal cord)	Two nt sequence types: In lung: 72.7% to gammaherpesviruses of harp seal (*Pagophilus groenlandicus*, KF466473, NC_055139) and ringed seal (*Pusa hispida*, KF466471, KF466472) In spleen: 73.4% to an alphaherpesvirus of American marten (*Martes Americana*, KX062131)	Two aa sequence types: In lung 75% to gammaherpesviruses of harp seal (NC_055139) and ringed seal (KF466471, KF466472) In spleen: 83.1% to an alphaherpesvirus of American marten (KX062131)	OQ926583	One sequence type, with 90.3% nt similarity to Mustelid gammaherpesvirus 3 (MN082680) found in European mink (*Mustela lutreola*)	One sequence type, with 95.4% aa similarity to Mustelid gammaherpesvirus 3 (MN082680) found in European mink

Crab-eating raccoon (*P. cancrivorus*)	OQ980212	70.7% to Ursid gammaherpesvirus 1 (JX220982) from a sun bear (*Helarctos malayanus*) and to Ursid gammaherpesvirus 2 found in an Asiatic black bear (*Ursus thibetanus*) MK089801 58.3% and 63.2% to gammaherpesvirus sequences found in the kidney and spinal cord of a South American coati of this study, respectively	76.2% to Ursid gammaherpesvirus 1 (MN685121, MN685127, MN685133) from sun bears 64.1% and 62.5% to gammaherpesvirus sequences found in the kidney and spinal cord of a South American coati of this study, respectively	OQ926578	78.9% Ursid gammaherpesvirus 2 found in an Asiatic black bear (MK089801) 63.1% to a gammaherpesvirus sequence found in several tissues of a South American coati of this study	92.0% Ursid gammaherpesvirus 2 found in an Asiatic black bear (MK089801) 67.5% to a gammaherpesvirus sequence found in several tissues of a South American coati of this study

South American coati (*N. nasua*)	OQ980213 (in spinal cord) OQ980214 (in kidney)	Two nt sequence types: In spinal cord: (87% to gammaherpesviruses described in pinnipeds (in ringed seals *(P. hispida* KF466472, KF466471); hooded seal *(Cystophora cristata* KF466474), and harp seal (NC_055139)) In kidney: 66.3% to a gammaherpesvirus found in Rickett's big-footed bat (*Myotis ricketti* JN692429)	Two aa sequence types: In spinal cord: 88% to gammaherpesviruses described in pinnipeds (in ringed seals (KF466472, KF466471); hooded seal (KF466474), and harp seal (NC_055139)) In kidney: 64% to ursid gammaherpesvirus found in sun bears (MN685121, MN685127)	OQ926579	One sequence type, with 89.8% nt similarity to harp seal herpesvirus found in a harp seal (NC_055139)	One sequence type, with 95.4% aa similarity to harp seal herpesvirus found in a harp seal (NC_055139)

ND = not detected.

## Data Availability

All data are available in the manuscript; the sequences obtained in this study are available at the public database GenBank under accessions numbers OQ926578–OQ926584 and OQ980212–OQ980218.

## Supplementary Materials

Supplementary Materials Table S1: detailed information about the herpesvirus-positive carnivores. Table S2: description of total prevalence and 95% confidence interval of herpesvirus in the whole study sample size according to the independent variables of sex, age, body condition, season, activity, and diet.
