# Supplementary material for: Novel Alpha-, Beta-, and Gammaherpesviruses in Neotropical Carnivores of Brazil
Source: Transbound Emerg Dis. 2024 Jun 6;2024:1347516. doi: 10.1155/2024/1347516 (PMC12020407; doi:10.1155/2024/1347516)
Supplement: Supplementary Materials — Table S1: detailed information about the herpesvirus-positive carnivores. Table S2: description of total prevalence and 95% confidence interval of herpesvirus in the whole study sample size according to the independent variables of sex, age, body condition, season, activity, and diet. [file 1347516.f1.docx]

**SUPPLEMENTARY TABLES**

**Supplementary Table 1.** Detailed information about the herpesvirus-positive carnivores.

| **ID** | **Family** | ***Scientific name*** | **Common name** | **Sex** | **Age class** | **Nutritional condition** | **Origin** | **Tested tissues** | **DPOL** | **gB** |
| --- | --- | --- | --- | --- | --- | --- | --- | --- | --- | --- |
| RK-303 | Felidae | *Leopardus guttulus* | southern tiger cat | M | J | Good | São Paulo | spleen | - | - |
|  |  |  |  |  |  |  |  | lung | - | **+ (γ)** |
| RK-175 | Felidae | *Leopardus pardalis* | ocelot | F | A | Good | São Paulo | spleen | **+ (γ)** | **+ (γ)** |
|  |  |  |  |  |  |  |  | lung | **+ (γ)** | **+ (γ)** |
| RK-300 | Felidae | *Puma yagouaroundi* | jaguarundi | M | A | Good | São Paulo | spleen | - | - |
|  |  |  |  |  |  |  |  | lung | - | **+ (γ)** |
| RK-012 | Mustelidae | *Galictis cuja* | lesser grison | M | J | Regular | São Paulo | spleen | - | - |
|  |  |  |  |  |  |  |  | lung | - | **+ (γ)** |
| RK-284 | Mustelidae | *Galictis cuja* | lesser grison | M | A | Good | São Paulo | spleen | - | - |
|  |  |  |  |  |  |  |  | lung | **+ (β)** | - |
| RK-035 | Mustelidae | *Lontra longicaudis* | neotropical river otter | M | A | Good | São Paulo | spleen | **+ (α)** | - |
|  |  |  |  |  |  |  |  | lung | **+ (γ)** | **+ (γ)** |
|  |  |  |  |  |  |  |  | mesenteric lymph node | - | - |
| RK-010 | Procyonidae | *Procyon cancrivorus* | crab-eating raccoon | F | J | Good | São Paulo | lung | - | **+ (γ)** |
| RK-037 | Procyonidae | *Procyon cancrivorus* | crab-eating raccoon | M | A | Good | São Paulo | spleen | - | **+ (γ)** |
|  |  |  |  |  |  |  |  | pancreas | - | - |
|  |  |  |  |  |  |  |  | tongue | - | - |
|  |  |  |  |  |  |  |  | Trachea | - | **+ (γ)** |
|  |  |  |  |  |  |  |  | ileum | - | - |
|  |  |  |  |  |  |  |  | liver | - | - |
|  |  |  |  |  |  |  |  | diaphragm | - | - |
|  |  |  |  |  |  |  |  | esophagus | **+ (γ)** | **+ (γ)** |
| RK-085 | Procyonidae | *Procyon cancrivorus* | crab-eating raccoon | M | A | Good | São Paulo | spleen | - | **+ (γ)** |
|  |  |  |  |  |  |  |  | lung | - | **+ (γ)** |
| RK-179 | Procyonidae | *Procyon cancrivorus* | crab-eating raccoon | M | A | Good | São Paulo | spleen | **+ (γ)** | **+ (γ)** |
|  |  |  |  |  |  |  |  | lung | **+ (γ)** | **+ (γ)** |
| RK-208 | Procyonidae | *Procyon cancrivorus* | crab-eating raccoon | M | J | Good | São Paulo | spleen | **+ (γ)** | **+ (γ)** |
|  |  |  |  |  |  |  |  | lung | **+ (γ)** | **+ (γ)** |
| RK-277 | Procyonidae | *Procyon cancrivorus* | crab-eating raccoon | F | A | Good | São Paulo | spleen | - | - |
|  |  |  |  |  |  |  |  | lung | **+ (γ)** | **+ (γ)** |
| RK-312 | Procyonidae | *Procyon cancrivorus* | crab-eating raccoon | M | J | Good | São Paulo | spleen | - | **+ (γ)** |
|  |  |  |  |  |  |  |  | kidney | - | **+ (γ)** |
|  |  |  |  |  |  |  |  | stomach | - | - |
|  |  |  |  |  |  |  |  | small intestine | - | **+ (γ)** |
|  |  |  |  |  |  |  |  | heart | **+ (γ)** | **+ (γ)** |
|  |  |  |  |  |  |  |  | spinal cord | **+ (γ)** | **+ (γ)** |
| RK-410 | Procyonidae | *Procyon cancrivorus* | crab-eating raccoon | F | A | Good | Santa Catarina | spleen | - | **+ (γ)** |
|  |  |  |  |  |  |  |  | liver | - | **+ (γ)** |
|  |  |  |  |  |  |  |  | brain | **+ (γ)** | **+ (γ)** |
|  |  |  |  |  |  |  |  | heart | - | **+ (γ)** |
|  |  |  |  |  |  |  |  | kidney | **+ (γ)** | **+ (γ)** |
|  |  |  |  |  |  |  |  | intestine | - | **+ (γ)** |
|  |  |  |  |  |  |  |  | lung | - | **+ (γ)** |
| RK-314 | Procyonidae | *Nasua nasua* | South American coati | F | A | Good | Paraná | spleen | - | - |
|  |  |  |  |  |  |  |  | kidney | **+ (γ)** | **+ (γ)** |
|  |  |  |  |  |  |  |  | heart | - | **+ (γ)** |
|  |  |  |  |  |  |  |  | stomach | - | **+ (γ)** |
|  |  |  |  |  |  |  |  | small intestine | - | **+ (γ)** |
|  |  |  |  |  |  |  |  | spinal cord | **+ (γ)** | **+ (γ)** |

**Supplementary Table 2**. Description of total prevalence and 95% confidence interval of herpesvirus in the whole study sample size according to the independent variables of sex, age, body condition, season, activity, and diet.

|  | **Sex** | | **Age** | | **Body condition** | | **Season** | | **Activity** | | **Ambient** | | | **Diet** | |
| --- | --- | --- | --- | --- | --- | --- | --- | --- | --- | --- | --- | --- | --- | --- | --- |
|  | Male | Female | Juvenile | Adult | Good | Regular | Rain | Dry | Noctural | Bothl | Field | Forest | River | Carnivore | Omnivore |
| **N** | 36 | 16 | 17 | 35 | 44 | 70 | 32 | 20 | 41 | 11 | 19 | 32 | 1 | 26 | 27 |
| **Prevalence** | 27.8 | 31.25 | 29.41 | 28.57 | 31.82 | 14.29 | 37.50 | 15.00 | 24.39 | 45.45 | 10.53 | 37.50 | 100.00 | 23.08 | 33.33 |
| **Lower 95% CI** | 12.41 | 5.74 | 5.26 | 12.83 | 17.49 | -0.2067 | 19.77 | -0.02146 | 10.67 | 10.37 | -4.67 | 19.77 | 0.00 | 5.72 | 14.33 |
| **Upper 95% CI** | 43.15 | 100.57 | 53.56 | 44.32 | 46.14 | 49.24 | 55.23 | 32.15 | 38.11 | 80.54 | 25.72 | 55.23 | 0.00 | 40.43 | 52.34 |
